# DIFFERENCES IN END-OF-LIFE OUTCOMES BETWEEN IMMIGRANT AND NONIMMIGRANT OLDER ADULTS

**DOI:** 10.1093/geroni/igad104.1845

**Published:** 2023-12-21

**Authors:** Zainab Suntai, Edson Chipalo, Susanny Beltran

**Affiliations:** Baylor University, Waco, Texas, United States; Lewis University, Romeoville, Illinois, United States; University of Central Florida, Orlando, Florida, United States

## Abstract

Although the number of immigrants in the United States has continued to grow, few research studies have focused on the experiences of older immigrants at the end life. Research on the immigration experience shows that there are long-lasting physical, mental, and psychological symptoms that continue to affect immigrants even long after arriving in a new country. To better understand how the immigration experience may influence outcomes in late life, this study examined differences between immigrant and non-immigrant older adults in the following end-of-life outcomes: being alone at death, being in pain at the end of life, having anxiety and/or depression in their final months of life, and the quality of care received at the end of life. Data were taken from the combined 2012 to 2020 National Health and Aging Trends study, which is an annual longitudinal study of Medicare beneficiaries aged 65 and older. Chi-square tests were used for bivariate analysis, and multivariate logistic regression models were used to assess differences between immigrant and non-immigrant older adults. The results showed that immigrant older adults were more likely to die alone at the end of life, more likely to be in pain at the end of life, more likely to experience anxiety and/or depression at the end of life, and less likely to receive good quality care at the end of life. Implications for practice, policy, and research are provided.

